# Disaster Report - Petroleum Depot (HAZMAT) Fire and Mass-Casualty Event in Guinea—December 18, 2023

**DOI:** 10.1017/S1049023X25101623

**Published:** 2025-12

**Authors:** Tamar T. Bah, Sory Conde, Lancei Toure, Mohamed Lamine Diallo, Lonceny Conde, Elin A. Gursky

**Affiliations:** 1. USAID Global Health Security Agenda Advisor (FMR), Conakry, Guinea; 2. Field Epidemiology and Laboratory Training Program, Director of National Agency for Health Security (ANSS), Ministry of Health of Guinea, Conakry, Guinea; 3. Director General of National Agency for Emergency Catastrophe and Humanitarian Crisis Management (ANGUCH), Kaloum, Conakry, Guinea; 4. Doctor Colonel, Director of Medical Rescue, Directorate General of Civil Protection, Ministry of Security and Civil Protection, Conakry, Guinea; 5. Program Coordinator, Guinean Red Cross, Conakry, Guinea; 6. Assist Professor of Clinical Sciences, William Carey University, College of Osteopathic Medicine, Hattiesburg, Mississippi, USA

**Keywords:** data management, disaster management, hazardous materials (HAZMAT), mass casualty incidents, West Africa

## Abstract

A chemical explosion and fire erupted in Conakry, Guinea, West Africa on December 18, 2023, destroying Guinea’s main fuel depot and resulting in 25 dead and 459 injured. Fifteen of the deaths occurred directly at the explosion site. Firefighters initiated efforts to control the blaze and transported injured, non-ambulatory victims to local hospitals with assistance from the military, Red Cross, and mining companies. Thirteen clinical facilities within an eight-mile radius of the explosion received burn and non-burn victims, with only one of these, Donka National Hospital, capable of handling burn victims. Many less seriously injured victims self-selected where they sought care, although anecdotal information indicates that an unknown number of injured did not seek care or chose to leave the city. The disaster marked the first time stakeholders from various sectors in the Guinean society (from first responders to mining companies) came together in a concerted response. Ranked 179th of 193 countries on the Human Development Index (HDI), the disaster rapidly outstripped Guinea’s response and health care capabilities, leaving behind economic shocks affecting livelihoods and the local economy. These experiences underscore the need for improved capabilities and coordination in disaster planning, warning and communication systems, and prehospital and hospital response in developing countries.

## Introduction

**Figure 1.**
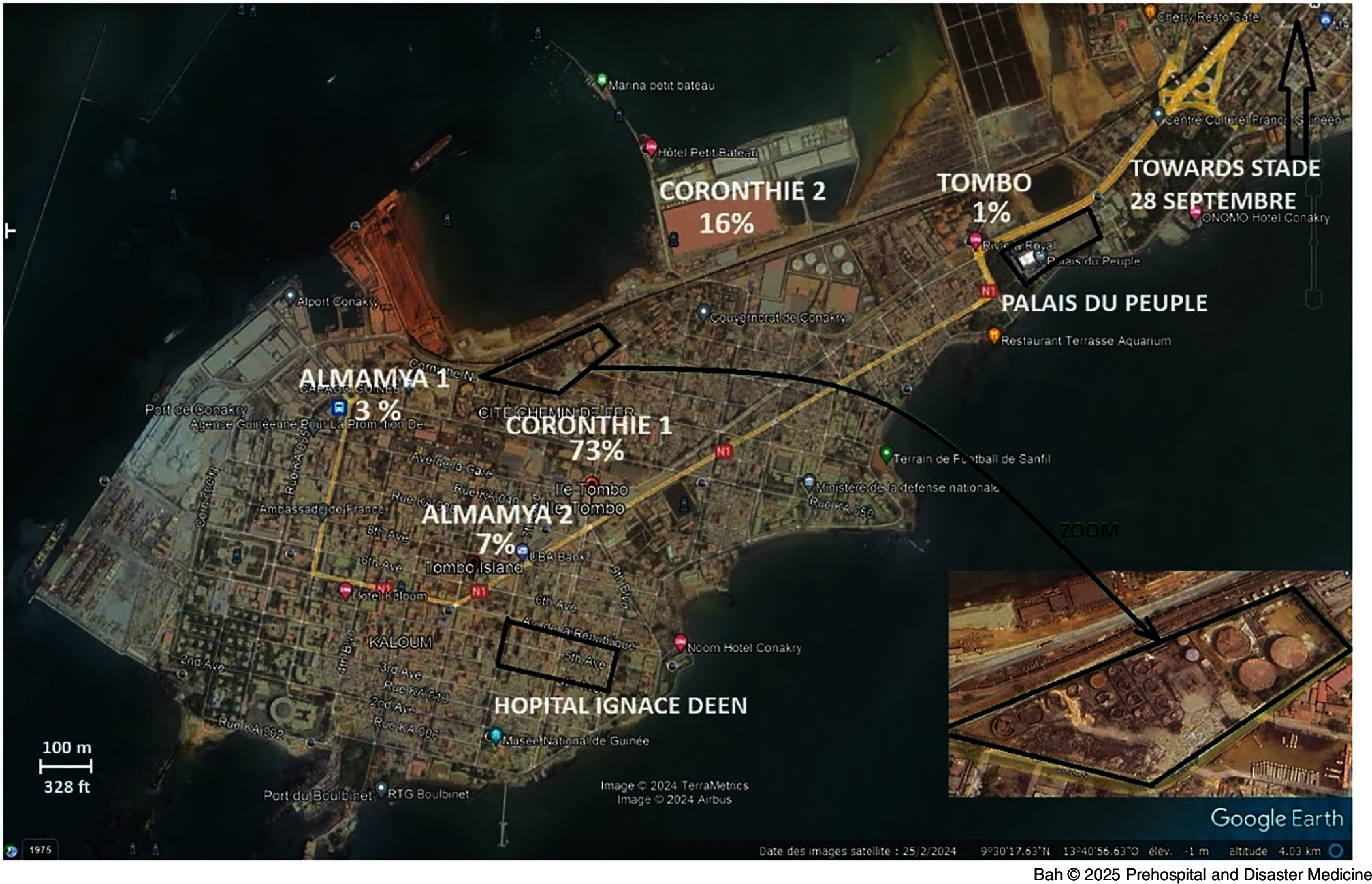
Google Earth Map of the Explosion Site in the Kaloum Peninsula.^a^ Note: The map shows distribution of casualties within residential districts, fuel depot, and relative locations of depot fuel tanks (trapezoid shape in Kaloum Peninsula, also enlarged, bottom right), Ignace Deen (bottom left rectangle) closest hospital to explosion site, and three mass-gathering sites; Palais Du Peuple (top left rectangle), September 28 Stadium (arrow top right), and Fayçal Mosque (not shown in the map but equidistant between the two). The distance between the explosion site and the three gathering sites is one-half mile, two miles, and three miles, respectively. ^a^Google Earth (Google Inc.; Mountain View, California USA).

An explosion and petrochemical fire struck Conakry, the capital of the Republic of Guinea (population 14.5 million) in West Africa^1^ on December 18, 2023. At its pre-fire level, the forty-year-old depot, the only such facility located within Guinea, contained the major part (10.5 million gallons) of the country’s supply of gasoline, jet fuel, and lubricant. In addition to the depot’s destruction, the explosion damaged the terminal’s offloading pipeline (an approximately 980ft segment) and surrounding administrative, commercial, and residential buildings. Damage was most extensive in Conakry’s residential districts of Kaloum,^2^ as shown on Figure 1, with Coronthie 1 at 73% and Coronthie 2 at 16% of homes destroyed, followed by Almamya at ten percent and Tombo at one percent.^3^ The aim of this report is to present a large-scale, multi-hazard incident in a limited resource setting. Information was first gathered from key actors during the response, then a comparison of similar events in Sub-Saharan Africa (SSA) was undertaken, and finally, observations and recommendations regarding chemical incident preparedness were provided.

The Kaloum peninsula covers a 1.3-square-mile area. The force of the explosion damaged several buildings within a three-to-four-mile radius and was felt 24 miles away.^4^ Fire and dense black smoke persisted for over one week, the duration of time required to fully extinguish the fire.^5^ Populations as far as 50 miles away reported seeing smoke, which was accompanied by strong fuel odors, oil droplets, particulate matter, and low night visibility.

Conakry’s population has grown since its independence from France in 1958, from 87,000 to 2.17 million people in 2024.^6^ This accelerating influx over the past 70 years has contributed to a more than 20-fold increase in population density proximate to Conakry’s port and oil depot, which also constitutes Guinea’s primary business and administration area. While the cause of the explosion remains unknown,^7^ its occurrence around midnight may have contributed to a lower number of deaths and injuries than had this explosion and fire taken place during daytime hours.

Thirteen clinical facilities within an eight-mile radius of the explosion received burn and non-burn victims. Twenty-eight burn victims were subsequently transferred to Donka National Hospital, the only facility in Guinea capable of handling burn patients. Many less seriously injured victims self-selected where they sought care or reportedly left the area. There was no central data repository for those who received prehospital or hospital care, which would facilitate characterizing the types and range of injuries associated with this disaster and would inform a strategy for managing medical follow up. Remains found at the explosionsite were placed in body bags. The government returned identified bodies to their families for ritual burials but was unable to identify an estimated 60% of remains.^8^

Guinea’s administration, business, and permitting centers in nearby Kaloum were rendered inoperable, constricting one of the world’s leading sources of Bauxite.^9^ To secure Guinea’s residual fuel supply, the government closed all gas stations and curtailed the movement of fuel trucks. Fuel was rationed to 25 liters per vehicle and five liters per motorcycle. Temporary emergency fuel supplies were negotiated with Sierra Leone, Senegal, and Cote d’Ivoire, however there were reports that some response groups had difficulty obtaining fuel sufficient to support their activities. Fuel restrictions led to the closure of schools and impacted livelihoods, trade, and other sectors.

## Source

This disaster report used multiple methods for the collection and analysis of quantitative and qualitative data.^10^ Both government and non-governmental/voluntary agencies were involved at various phases of the response and in the collection of information regarding issues pertaining to health, food, and safety. Key informant interviews sought to assemble the disaster experiences of indigenous stakeholders that included Guinea’s Ministries of Health and Public Hygiene, Security and Civil Protection, Social Affairs and the Advancement of Women and Children, and the Guinean Red Cross (Conakry, Guinea).

The National Agency for Emergency Catastrophe and Humanitarian Crises Management (Agence Nationale de Gestion des Urgences et Catastrophes Humanitaires [ANGUCH]; Kaloum, Conakry, Guinea) provided twice daily in-person, multi-sector updates. Red Cross volunteers went door-to-door to collect information. Data were compiled at the end of the day and presented for review by ANGUCH. These updates were synthesized to help inform this manuscript.

No information on identifiable people was collected. Quantitative information enumerating the number and types of casualties was extracted by hand from paper and electronic medical records (where available) from all clinical facilities (Table 1) by ward clerks and other personnel and subsequently input into Excel spreadsheets (Microsoft Corp.; Redmond, Washington USA). This information was later grouped into diagnostic categories (Table 2) by a medical doctor (Author #2).

**Table 1.** Number of Casualties Received per Health Facility and Hospital

Name of Health Facilities	Number of Casualties
Army Health Services	30
Central Prison	35
Clinic Ambroise	8
Clinic Pasteur	6
District Health Facility Coleah	15
District Health Facility Flamboyant	1
District Health Facility Koulewondy	3
District Health Facility Matam	4
District Health Facility Ratoma	15
Donka Hospital	135
Ignace Deen Hospital	79
Sino-Guinean Hospital	10
Sports Medical Center of September 28 Stadium	118
Total	459

Note: Listed alphabetically; collected by ANGUCH Health Commission January 02, 2024.

Abbreviation: ANGUCH, National Agency for Emergency Catastrophe and Humanitarian Crisis Management.

**Table 2.** Conditions Recorded on Patient Charts for Individuals Admitted from December 20 - 28, 2023 Across All Hospitals and Facilities.

Category	Condition(s) Recorded on Patient Charts	Number
Contusion	Contusion	148
Brain and Spinal Cord	Skull Trauma, Simple Trauma, Vertebral Trauma, Dizziness, Headache, Notion of Loss of Consciousness, Hearing Disorder, Fainting	33
Burns	Burns	28
Cardiopulmonary	Respiratory Disorders, Dyspnea, Chest Pain, Suffocation, Myocardial Infarction, Palpitations, Prostration	19
Fracture	Fracture, Polytraumatized	9
Other, Non-Specific	Physical Asthenia, Notion of Fever, Emotional Hypertension, Pharyngitis, Fever, Cough, Adnexitis, Anorexia, Body Pain	84
Abdominal	Abdominal Pain, Epigastric Syndrome, Vomiting	1
TOTAL		322

Note: Conditions displayed in descending order of frequency.

All collected data were integrated and displayed using graphic and visual presentation, descriptive statistics, and qualitative methods to illuminate gaps in capabilities, coordination, and information capture and analysis within and across the health, disaster response, and non-governmental sectors. This manuscript was reviewed in French and approved by the health research ethical review board of Guinea, response No 014/CNERS/25.^11^

## Observations

The ANGUCH final report lists 11,074 people in 2,141 households affected by the explosion and petrochemical fire, of which 18% were children under five years of age.^3^ Included among the identified dead and seriously injured were fuel depot workers and guards, night-shift port rotation staff, and homeless or otherwise displaced persons whose post-event status is unknown. Incomplete and inconsistent reporting resulted in conflicting numbers of deaths and injuries. As more data became available, the number of casualties increased to 25 deaths and 459 injured, although these figures may be under-estimated. (Table 2)

Three major observations can be seen from the disaster response in Guinea.

### Lesson One: Prepare Medical Services for Mass-Casualty Events

Over 780 people came to the Emergency Operations Center, which was a 1,500 square meter tent at the Palais du Peuple,^12^ set up within the first sixteen hours following the explosion. Approximately 400 people, mostly women and children, were prioritized for sheltering in the tent for 48 hours. The facility was relocated to Guinea’s oldest sports stadium^13^ a few days later to facilitate security and coordination of services. In total, ANGUCH estimated that over 1,000 people fled the city.

Enumerating and classifying the numbers and types of injuries sustained from the fire and petrochemical explosion was severely constrained by a lack of consistent and reliable information collection within and across Guinea’s hospitals and response agencies. With the exception of Donka Hospital, which upgraded to electronic medical records in 2022, most hospitals rely upon paper data collection methods. Measuring the clinical impact of the explosion was further impeded by the absence of an injury and disease classification system, which had not been incorporated into Guinea’s health system.

### Lesson Two: Limit Population Growth in Proximity to Hazardous Sites

Like many countries in SSA, Guinea has no domestic refining capacity and relies entirely on imported fuels that arrive by tanker ship, are transferred to large storage depots, and then redistributed by trucks to retail sites. Each point in this supply and storage chain is vulnerable to aging infrastructure, inconsistent oversight, and in some settings, deliberate theft, tampering, and other forms of disruption.^14,15^ As a single point of failure, disruption to the global supply of Bauxite, business and education magnified the toll of injury and death sustained by Guinea.^16^

While Guinea’s fuel storage capacity was leveraged to support the country’s economic growth and population expansion, the latter of which was generally proximate to the depot, concomitant growth of hospital and disaster response systems were not similarly addressed to provide threat mitigation.

### Lesson Three: Monitor and Maintain Disaster Response Capacity

Guinea’s fragile public health system was particularly strained by this petrochemical disaster and mass-casualty event. This reinforces the assessments by the World Health Organization (WHO; Geneva, Switzerland) Joint External Evaluation (JEE) in February 2017 and May 2023, noting Guinea’s “non-existent capacity” for responding to chemical events, resulting in the lowest assessment score possible (one out of five). The JEE further noted, “Regarding preparedness for major public health events, it is evident that Guinea lacks policies, strategies, or national plans for the detection, assessment, and response to radiological and chemical emergencies.”^17,18^

When this event occurred in December 2023, the Guinean government had no formalized incident command structure in place to lead multi-domain responders or to redistribute fire suppression resources in the event of an escalating or concurrent emergency. Guinea’s five-ambulance fleet was quickly overwhelmed and augmented by a variety of government and private vehicles.^19^ Among the almost 300-person firefighting workforce, many lacked formal training in fire suppression for petrochemical response protocols befitting the scale and complexity of this event, and some sustained burns and respiratory system injuries.^19^ Few responders were provided with protective body and respiratory gear.^19^ Two days following the explosion, a medical aid station was stood up to triage and provide care to firefighters who had sustained burns and other explosion- and fire-related injuries. The most serious cases were evacuated to the military hospital.^19^

## Analysis

Sub-Saharan African countries depend on a complex, cross-border, liquid-fuel network of depots, overland transport, and limited pipelines—much of it aging, under-regulated, and chronically under-invested.^20^ Rapid urban growth, from roughly 27% in 1990 to nearly 47% in 2023, has intensified population exposure to industrial hazards and legacy infrastructure.^21^ While countries with higher Human Development Index (HDI; United Nations Development Program; New York USA) scores generally benefit from stronger regulatory oversight and basic redundancies, risk profiles across the region remain highly uneven.^22^

Limited health and emergency response systems in SSA are not unique to Guinea.^23^ Between 2016 and 2019, 40 out of 47countries participated in the JEE.^24^ The findings showed that under the International Health Regulations (IHR; WHO) capacity “respond,” there were critical gaps in multi-hazard public health emergency preparedness. For chemical and radiation emergencies, most countries in SSA scored at Level 1 or Level 2, indicating minimal or no capacity.^24^ The second round of JEE, started in 2022, indicated that although certain countries in SSA improved their capacities in surveillance and detection areas, there are persisting gaps in response capabilities for chemical events, as indicated by scores between one or two.^25^

Mass-casualty fuel and chemical events similar to the 2023 Guinea explosion have recurred across the past decade, including the Morogoro tanker disaster in Tanzania (2019), an explosion in Cap-Haitien, Haiti (2021), and the Boksburg, South Africa incident (2022).^26–28^ Although the Conakry event was more limited in scale—possibly because it occurred at midnight rather than during peak commercial activity—it revealed the degree to which industrial accidents can precipitate cascading public-health and systems-level consequences in low-resource settings. Fuel scarcity, school and business closures, and disruptions to Guinea’s Bauxite sector produced measurable downstream effects that extended far beyond the immediate blast zone.^29^ For emergency planners, these impacts underscore the need to treat depot and transport-system failures not as isolated industrial mishaps, but as high-consequence events with national and regional implications for health security.

Since the Kaloum explosion, ANGUCH has strengthened its coordination role, and Guinea has adopted a National Multi-Hazard Contingency Plan across government and private sector partners. Relocating the depot to a less densely populated area, mapping at-risk infrastructure, and improving community alert systems are meaningful steps toward integrated risk reduction. For countries where low-incidence, high-impact disasters compete with urgent day-to-day priorities, investment in disaster response offers dual benefit opportunities to reduce population vulnerability.

At the regional level, collective capability can be built through shared firefighter and prehospital training, pooled equipment, and interoperable ambulance systems. Memoranda of Agreement can leverage economies of scale in burn and trauma care, surge capacity, and response speed. Such efforts directly advance the core aims of disaster medicine: strengthening preparedness, protecting frontline responders, and reducing preventable morbidity and mortality during complex industrial events.

Finally, within a public-safety and health-security framework, engagement with the petrochemical sector can motivate depot relocation, pipeline reinforcement, and joint financing of risk-reduction initiatives as essential protections for surrounding communities and critical services. Collaborative efforts between government and private industry will build robust and resilient systems better able to recover from the types of fuel-related disasters that continue to challenge health systems across SSA.
